# Sense of purpose and internet gaming disorder symptoms among college students: a cross-sectional moderated mediation analysis

**DOI:** 10.3389/fpsyg.2026.1819780

**Published:** 2026-10-22

**Authors:** Tianran Li, Xiaorui Mao, Yuwei Wang

**Affiliations:** 1 School of Education, Beijing City University, Beijing, China; 2 Psychological Counseling Centre, Beijing City University, Beijing, China; 3 School of Psychology, Shaanxi Normal University, Xi'an, Shaanxi, China

**Keywords:** college students, internet gaming disorder, moderated mediation analysis, self-control, sense of purpose

## Abstract

**Introduction:**

Internet Gaming Disorder (IGD) symptoms are a concern among college students, but the cross-sectional associations among sense of purpose, self-control, and IGD symptoms remain unclear.

**Objective:**

This study examined the statistical indirect association between sense of purpose and IGD symptoms through self-control and whether gender moderated this indirect association.

**Methods:**

A cross-sectional survey included 1,210 Chinese college students (44.5% male; mean age = 20.00 years, SD = 1.64). Participants completed measures of IGD symptoms (9 items, Cronbach’s *α* = 0.949), sense of purpose (14 items, *α* = 0.945), and self-control (19 items, *α* = 0.892). Age-adjusted PROCESS Models 4 and 14 were estimated using 5,000 bootstrap samples.

**Results:**

Sense of purpose was positively associated with self-control (*B* = 0.403, *p* < 0.001) and negatively associated with IGD symptoms before self-control was included in the model (*B* = −0.147, *p* < 0.001). The statistical indirect association through self-control was significant [*B* = −0.205, 95% bootstrap CI (−0.249, −0.161)], whereas the direct association after accounting for self-control was positive (*B* = 0.058, *p* = 0.006). The opposite signs of the indirect and direct coefficients indicated an inconsistent mediation or statistical suppression pattern. Gender moderated the association between self-control and IGD symptoms (*B* = 0.107, *p* < 0.001). Conditional indirect associations were −0.224 [95% bootstrap CI (−0.273, −0.175)] for males and −0.181 [95% bootstrap CI (−0.222, −0.141)] for females; the index of moderated mediation was 0.043 [95% bootstrap CI (0.017, 0.070)].

**Conclusion:**

In this cross-sectional sample, sense of purpose was negatively associated with IGD symptoms, and a significant negative statistical indirect association through self-control was observed. This indirect association was stronger among male than female students. These findings describe cross-sectional statistical relationships and should not be interpreted as evidence of temporal or causal mechanisms.

## 1 Introduction

In the digital age, online gaming has become ubiquitous and represents an important form of daily entertainment for millions worldwide. However, loss of control over gaming can have serious negative consequences, leading to Internet Gaming Disorder (IGD), a condition that has garnered increasing attention from mental health professionals and researchers. IGD has been included in the fifth edition of the Diagnostic and Statistical Manual of Mental Disorders (DSM-5) due to its significant mental health implications and the growing body of evidence demonstrating its detrimental effects (Ko, 2014). Its core features include an inability to control gaming use, which leads to substantial impairment in psychological, social, and academic functioning, affecting individuals’ daily lives and overall wellbeing (Gentile et al., 2017). College students represent a particularly high-risk population for developing IGD due to factors such as increased autonomy, reduced parental supervision, easy access to gaming platforms, and the stress associated with academic demands. Relevant surveys indicate that the overall incidence of internet addiction among college students is approximately 5%, with notably higher rates among male students, generally around 10% (Borges et al., 2019; Liu et al., 2022). Other research indicates that college students with internet addiction commonly experience a range of mental health problems such as decreased self-esteem, insufficient social efficacy, increased loneliness, academic burnout, social withdrawal, substance addiction, and alcohol addiction (Baturay and Toker, 2019; Schmitt and Livingston, 2015). Therefore, studying how to effectively prevent and control IGD among college students is of great significance for promoting their mental health, enhancing their academic progress, and ensuring their successful transition into adulthood.

### 1.1 The relationship between sense of purpose and internet gaming disorder

Sense of purpose is a relatively stable orientation toward long-term aims that are meaningful to the self and consequential beyond the self (Damon et al., 2003). It can organize priorities and provide a basis for evaluating whether immediate activities are consistent with longer-term goals. Prior longitudinal evidence has linked purpose in life to lower subsequent IGD symptoms among Chinese university students (Zhang et al., 2019). The present study therefore examines whether the same variables are associated in a cross-sectional college sample.

A stronger sense of purpose may be associated with fewer IGD symptoms because clear long-term aims can make conflicts between immediate gaming rewards and valued goals more salient. This rationale does not by itself establish temporal or causal direction. Hypothesis 1 therefore proposes a negative cross-sectional association between sense of purpose and IGD symptoms.

### 1.2 The proposed indirect association through self-control

Self-regulation theories propose that goal pursuit depends not only on identifying valued goals but also on regulating behavior in the presence of competing impulses (Gollwitzer, 1999; Little et al., 2007). Self-control may therefore provide one statistical link between sense of purpose and IGD symptoms: purpose reflects the orientation of behavior toward meaningful aims, whereas self-control reflects the capacity to act consistently with those aims when gaming offers immediate rewards.

Sense of purpose and self-control are theoretically related but non-equivalent constructs. Sense of purpose concerns the identification, meaningful experience, and regulation of long-term goals, whereas self-control concerns the capacity to inhibit impulses and align behavior with standards across situations. Thus, the goal-regulation component of purpose specifies how goals are organized and monitored, while self-control reflects a broader behavioral capacity for implementing those goals when immediate temptations compete with them. In the present cross-sectional model, self-control is therefore treated as a distinct statistical mediator linking goal-directed orientation to IGD symptoms, while the possibility of partial conceptual overlap is evaluated empirically and considered in sensitivity analyses.

Self-control, as a key component of individual psychological functioning, refers to an individual’s ability to autonomously regulate their behavior to align with their internal value system and general social expectations, successfully inhibit impulses, resist external temptations, and achieve established goals (Baumeister, 2018). This capacity for behavioral regulation becomes especially crucial in the digital age, where immediate gratifications and readily available online entertainment constantly compete for individuals’ attention and time. Lower self-control has been consistently associated with higher levels of internet-related addictive behaviors, including gaming-related problems. Prior research has linked higher self-control with lower levels of maladaptive and impulsive behaviors (De Ridder et al., 2012). Several self-regulatory processes may help explain the association between higher self-control and fewer problematic behaviors, including greater resistance to immediate impulses and a greater capacity to redirect attention toward goal-consistent activities. Furthermore, self-control facilitates the implementation of specific situational strategies that help individuals avoid or manage high-risk situations where gaming temptations are particularly strong (Duckworth et al., 2016).

Specifically for college students, those with higher levels of self-control possess stronger delayed gratification abilities; they can restrain the current impulse to play games and allocate more valuable resources such as time and energy to important goal tasks related to future development (Duckworth et al., 2016). This capacity becomes particularly important during the university years when students face numerous competing demands and must balance immediate pleasures against long-term academic and career objectives. Students with robust self-control can more effectively prioritize their time, maintaining appropriate boundaries around gaming activities while ensuring adequate attention to academic responsibilities, social relationships, and personal development goals. A series of studies has found that students with strong self-control ability exhibit less internet addiction behavior (Li et al., 2021) and less IGD symptoms (Safarina and Halimah, 2019). These findings provide a rationale for examining self-control as a statistical correlate that may help account for the cross-sectional association between sense of purpose and IGD symptoms. Therefore, Hypothesis 2 proposes that self-control statistically mediates the cross-sectional association between sense of purpose and IGD symptoms.

### 1.3 The moderating role of gender

Gender differences have been reported in both problematic gaming and the association between self-control and internet-related addictive behaviors. Male students generally report higher levels of problematic gaming, and a meta-analysis found that the negative association between self-control and internet addiction was stronger in male samples (Li et al., 2021). These findings motivate a moderation hypothesis but do not identify the social or developmental processes responsible for the difference. Hypothesis 3 therefore proposes that gender moderates the association between self-control and IGD symptoms, with a stronger negative association among male students.

## 2 Materials and methods

### 2.1 Participants

A cluster convenience sample of college students completed an online questionnaire. Recruitment was coordinated primarily through the psychological counseling center and course instructors across several colleges at Beijing City University; a small number of students from other universities also participated. A total of 1,210 completed questionnaires were received, and all were retained for analysis; no completed questionnaires were excluded. The sample comprised 672 female students (55.5%) and 538 male students (44.5%). No gamer-screening item was administered; all participants completed the IGD symptom items regardless of recent gaming status. Gender was coded as 1 = male and 2 = female. The mean age of participants was 20.00 years (SD = 1.64).

### 2.2 Measures

#### 2.2.1 Internet gaming disorder symptom items

The nine symptom items corresponded to the DSM-5 IGD criteria as operationalized by Petry et al. (2014), whose consensus-based assessment framework also provided multilingual translations, including Chinese. In the present survey, the Chinese item wording was adapted for use with college students, and responses were recorded on a five-point endorsement scale from 1 (very inconsistent) to 5 (very consistent). Similar five-point response adaptations of Petry-derived IGD symptom items have been used in previous Chinese samples (Wei et al., 2023; Ji et al., 2024), and psychometric research has also supported a Chinese polytomous version of the nine-item IGD measure (Lei et al., 2020). However, because the response anchors and 3-month timeframe used in the present study were not identical to those validated versions, the present measure is treated as an adapted IGD symptom index rather than a separately validated Chinese version of a named diagnostic scale. Participants were instructed to report their gaming-related experiences during the previous 3 months. The nine items were summed to produce a total score ranging from 9 to 45, with higher scores indicating greater IGD symptom severity. An example item is, “When trying to cut back or stop playing games, or when you are unable to play, do you feel restless, irritable, angry, anxious, or sad?” Cronbach’s α was 0.949 in the present sample.

#### 2.2.2 College students’ sense of purpose questionnaire

The College Students’ Sense of Purpose Questionnaire developed by Cai et al. (2024) measures sense of purpose among university students. The scale comprises three dimensions: goal recognition, goal experience, and goal regulation, with a total of 14 items. It employs a 5-point Likert rating system ranging from 1 (strongly disagree) to 5 (strongly agree), with one reverse-scored item. Higher total scores indicate stronger sense of purpose. Sample items include: “I am very clear about what my goals are at this stage” (goal recognition dimension) and “I believe that my goals are meaningful and worth pursuing” (goal experience dimension). The original scale demonstrated good reliability and validity (Cai et al., 2024). In the current study, Cronbach’s *α* was 0.945, indicating excellent internal consistency.

#### 2.2.3 College students’ self-control scale

The College Students’ Self-Control Scale revised by Tan and Guo (2008) assesses self-regulatory capacity among university students. This scale contains five dimensions: impulse control, healthy habits, resisting temptation, focused work, and moderate entertainment, with a total of 19 items. The scale employs a 5-point rating system ranging from 1 (does not describe me at all) to 5 (describes me very well), with higher scores indicating stronger self-control capacity. Sample items include: “I can work efficiently toward a long-term goal” (focused work dimension) and “I am good at resisting temptations” (resisting temptation dimension). In the current study, Cronbach’s α was 0.892, demonstrating good internal consistency. Although this measure includes dimensions related to goal-directed regulation, it assesses behavioral control across impulse, habit, temptation, work, and entertainment contexts rather than the meaning and organization of life goals assessed by the sense-of-purpose measure.

### 2.3 Statistical analysis

SPSS 26.0 was used for descriptive statistics and correlation analyses. PROCESS 3.0 Models 4 and 14 were used to estimate the indirect effect and second-stage moderated mediation, respectively. All reported PROCESS coefficients are unstandardized coefficients (B). Bootstrap confidence intervals were based on 5,000 resamples; an interval excluding zero was considered statistically significant. Age was included as a covariate in both models. Gender was coded 1 = male and 2 = female. For Model 14, self-control and gender were mean-centered before the interaction term was formed. Discriminant validity between sense of purpose and self-control was examined using the heterotrait-monotrait ratio of correlations (HTMT; Henseler et al., 2015). Sensitivity analyses re-estimated the age-adjusted indirect and moderated indirect effects after excluding self-control dimensions with the greatest potential content overlap with purpose.

## 3 Results

### 3.1 Common method bias test

All variables were assessed by self-report. Anonymous and confidential administration was used to reduce evaluation concerns. Harman’s single-factor test yielded five factors with eigenvalues greater than 1, and the first factor accounted for 22.49% of the variance. Because this diagnostic cannot rule out common-method bias, the result was treated only as a limited descriptive check.

### 3.2 Descriptive statistics and correlation analysis

Descriptive statistics and zero-order correlations are shown in Table 1. Sense of purpose was positively correlated with self-control and negatively correlated with IGD symptoms; self-control was also negatively correlated with IGD symptoms (all *p* < 0.001).

**Table 1:** Descriptive statistics and correlation analysis results.

Variable	M ± SD	1	2	3
1. Sense of purpose	51.733 ± 9.704	1		
2. Self-control	59.517 ± 12.838	0.307***	1	
3. IGD symptoms	21.603 ± 9.253	−0.158***	−0.690***	1

*p < 0.05, **p < 0.01, ***p < 0.001.

### 3.3 Discriminant validity

The HTMT value between sense of purpose and self-control was 0.356, below the conservative 0.85 criterion and therefore supporting empirical separability at the item-correlation level. This result does not eliminate the possibility of content overlap, which was examined further in the sensitivity analyses reported in Section 3.7.

### 3.4 Gender differences in sense of purpose, self-control, and IGD symptoms

Independent-samples t tests examined gender differences (Table 2). Sense of purpose did not differ significantly by gender (*t* = −0.149, *p* = 0.882). Female students reported higher self-control than male students (*t* = −2.422, *p* = 0.016), whereas male students reported higher IGD symptom scores than female students (*t* = 7.203, *p* < 0.001).

**Table 2:** Gender difference tests.

Variable	Male (*N* = 538)	Female (*N* = 672)	*t*	*p*
1. Sense of purpose	51.69 ± 10.44	51.77 ± 9.08	−0.149	0.882
2. Self-control	58.52 ± 13.27	60.32 ± 12.44	−2.422^*^	0.016
3. IGD symptoms	23.73 ± 9.64	19.90 ± 8.57	7.203^***^	<0.001

*p < 0.05, **p < 0.01, ***p < 0.001.

### 3.5 Statistical indirect effect analysis

PROCESS Model 4 was used to examine the statistical indirect effect of sense of purpose (X) on IGD symptoms (Y) through self-control (M), with age included as a covariate. Sense of purpose was positively associated with self-control [path a: *B* = 0.403, SE = 0.036, *t* = 11.101, *p* < 0.001, 95% CI (0.332, 0.474); *R*^2^ = 0.096]. Controlling for sense of purpose and age, self-control was negatively associated with IGD symptoms [path b: *B* = −0.510, SE = 0.016, *t* = −32.366, *p* < 0.001, 95% CI (−0.541, −0.479)]. The age-adjusted total association between sense of purpose and IGD symptoms was negative [path c: *B* = −0.147, SE = 0.027, *t* = −5.433, *p* < 0.001, 95% CI (−0.201, −0.094)], whereas the direct association after accounting for self-control and age was positive [path c′: *B* = 0.058, SE = 0.021, *t* = 2.779, *p* = 0.006, 95% CI (0.017, 0.099)]. Age was not independently associated with self-control (*B* = −0.280, *p* = 0.191) or IGD symptoms in the direct-effect model (*B* = 0.124, *p* = 0.293) (see Figure 1).

**Figure 1:**
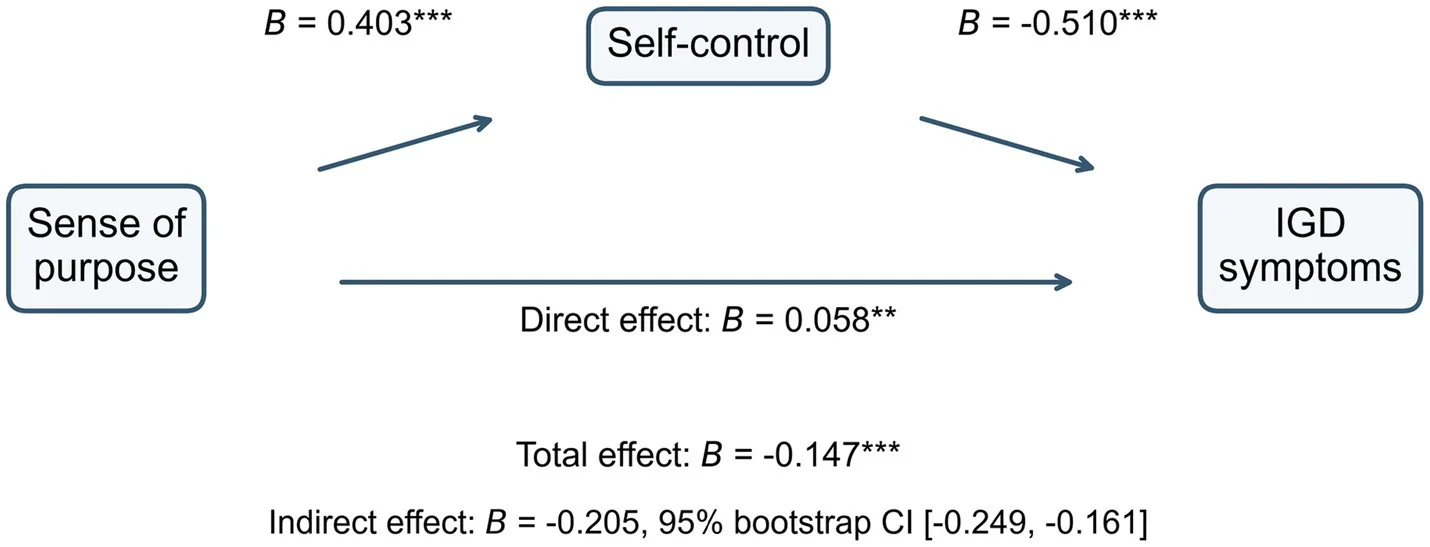
Age-adjusted cross-sectional statistical mediation model linking sense of purpose, self-control, and IGD symptoms. Values are unstandardized coefficients (B). **p < 0.01, ***p < 0.001.

The age-adjusted indirect effect through self-control was −0.205 [bootstrap SE = 0.022, 95% bootstrap CI (−0.249, −0.161)]. Because the negative indirect effect and positive direct effect had opposite signs, the result was classified as an inconsistent mediation or statistical suppression pattern rather than full or partial mediation (see Table 3).

**Table 3:** Analysis of the statistical indirect effect.

Effect	Effect value	SE	95%CI
Total effect	−0.147	0.027	[−0.201, −0.094]
Direct effect	0.058	0.021	[0.017, 0.099]
Indirect effect	−0.205	0.022	[−0.249, −0.161]

### 3.6 Moderated mediation analysis

PROCESS Model 14 tested whether gender moderated the second stage of the indirect association. Sense of purpose was the predictor, self-control the mediator, IGD symptoms the outcome, gender the moderator, and age a covariate in both model equations.

As shown in Table 4, sense of purpose was positively associated with self-control (*B* = 0.403, *p* < 0.001). The self-control × gender interaction was associated with IGD symptoms (*B* = 0.107, *p* < 0.001), indicating that the self-control-IGD symptom slope differed by gender. Age was not independently associated with self-control (*B* = −0.280, *p* = 0.191) or IGD symptoms (*B* = 0.117, *p* = 0.306).

**Table 4:** Moderated mediation analysis results.

Variable	Dependent variable: self-control			Dependent variable: IGD symptoms		
	B	*t*	*95%CI*	B	*t*	*95%CI*
Age	−0.280	−1.308	[−0.701, 0.140]	0.117	1.023	[−0.107, 0.341]
Sense of purpose	0.403	11.101***	[0.332, 0.474]	0.050	2.486*	[0.011, 0.090]
Gender				−2.904	−7.704***	[−3.644, −2.165]
Self-control				−0.497	−32.271***	[−0.527, −0.467]
Self-control × gender				0.107	3.637***	[0.049, 0.164]
*R* ^2^	0.096			0.509		
*F*	63.763***			249.844***		

*B*, unstandardized coefficient. Age was included as a covariate. Gender was coded 1 = male and 2 = female; self-control and gender were mean-centered before the interaction term was formed. *p < 0.05, **p < 0.01, ***p < 0.001.

Simple-slope estimates were negative for both groups. The age-adjusted association between self-control and IGD symptoms was stronger among males (*B* = −0.556, *p* < 0.001) than females (*B* = −0.449, *p* < 0.001; Figure 2).

**Figure 2:**
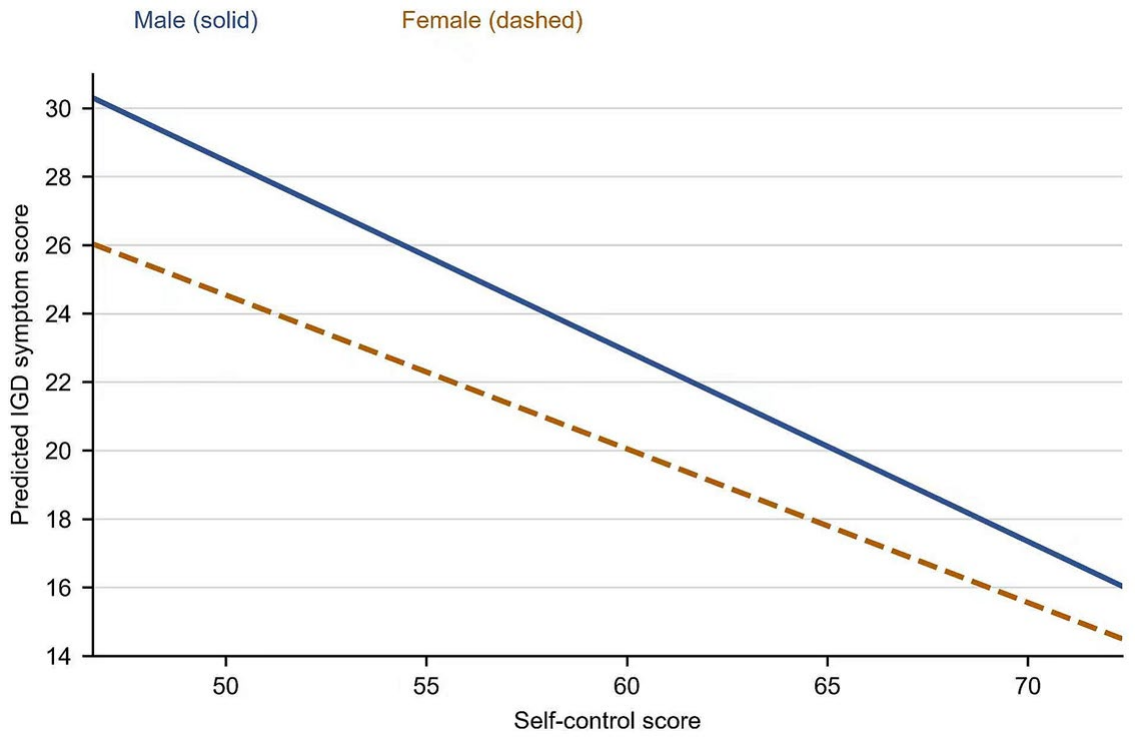
Gender moderation of the age-adjusted cross-sectional association between self-control and IGD symptoms. Predicted values are shown at the sample means of sense of purpose and age across ±1 SD of self-control.

The age-adjusted conditional indirect effect of sense of purpose on IGD symptoms through self-control was −0.224 for males [95% bootstrap CI (−0.273, −0.175)] and −0.181 for females [95% bootstrap CI (−0.222, −0.141]). The index of moderated mediation was 0.043 [bootstrap SE = 0.014, 95% bootstrap CI (0.017, 0.070)], supporting a gender difference in the statistical indirect effect.

### 3.7 Sensitivity analyses

Age-adjusted sensitivity analyses retained the same substantive conclusion when potentially overlapping self-control dimensions were excluded (Table 5). Across alternative mediator specifications, indirect effects ranged from −0.086 to −0.223, and indices of moderated mediation ranged from 0.014 to 0.053; every 95% bootstrap confidence interval excluded zero.

**Table 5:** Sensitivity analyses using alternative self-control specifications.

Mediator specification	Indirect effect [95% bootstrap CI]	Male conditional indirect effect [95% bootstrap CI]	Female conditional indirect effect [95% bootstrap CI]	Index of moderated mediation [95% bootstrap CI]
Full 19-item scale	−0.205 [−0.249, −0.161]	−0.224 [−0.273, −0.175]	−0.181 [−0.222, −0.141]	0.043 [0.017, 0.070]
Excluding resisting temptation and focused work	−0.091 [−0.137, −0.044]	−0.096 [−0.145, −0.046]	−0.082 [−0.124, −0.039]	0.014 [0.003, 0.029]
Impulse control and healthy habits only	−0.086 [−0.130, −0.040]	−0.092 [−0.139, −0.042]	−0.077 [−0.116, −0.035]	0.015 [0.004, 0.031]
Excluding moderate entertainment	−0.223 [−0.265, −0.181]	−0.247 [−0.296, −0.200]	−0.194 [−0.234, −0.154]	0.053 [0.025, 0.083]

Estimates are unstandardized and adjusted for age. Confidence intervals are based on 5,000 bootstrap resamples.

## 4 Discussion

### 4.1 Gender differences in IGD and self-control

Male students in our sample reported higher IGD symptom scores and lower self-control than female students. The gender difference in IGD symptoms accords with earlier findings among Asian adolescents and Chinese male college students (Ko et al., 2005; Liu et al., 2022). It also complements meta-analytic evidence that stronger self-control is associated with lower levels of problematic Internet use among students (Li et al., 2021). Together, these findings suggest that gender and self-regulatory capacity are relevant to the distribution of IGD symptoms, although mean differences do not explain the processes that produce them.

Several contextual interpretations remain plausible. Gender-related differences in preferred game activities, achievement and social motives, or the acceptability of intensive gaming may contribute to the observed pattern (Ko et al., 2005; Yee, 2006; Laconi et al., 2017). The Gendered Family Process Model also identifies differentiated socialization and parental expectations as one possible developmental context for gendered behavior (Endendijk et al., 2018). However, gaming motives, genre preferences, parenting, and peer norms were not measured here. These accounts should therefore be treated as competing hypotheses for future research, not as mechanisms established by our findings.

### 4.2 Association between sense of purpose and IGD symptoms

A stronger sense of purpose was associated with fewer IGD symptoms before self-control was entered into the model. This result converges with one-year longitudinal evidence showing that purpose in life predicted lower subsequent IGD symptoms among Chinese university students (Zhang et al., 2019). It is also consistent with research linking purpose to broader health and wellbeing during young adulthood (Chen et al., 2019). The convergence across outcomes supports the view that purpose is a potentially important psychological resource, while our cross-sectional estimate remains an association rather than evidence of a protective causal effect.

One interpretation is that meaningful long-term aims provide a standard against which immediate activities are evaluated. Purpose may make the opportunity costs of prolonged gaming more salient when gaming conflicts with academic, relational, or developmental goals (Damon et al., 2003; Kang et al., 2019). Research on implementation intentions further suggests that translating valued goals into concrete plans can reduce the gap between intention and behavior (Gollwitzer, 1999). This account remains provisional because goal implementation, gaming time, and perceived goal conflict were not measured. The reverse pathway is also possible, as persistent gaming-related impairment could weaken progress toward valued goals and, in turn, diminish a person’s sense of purpose.

### 4.3 Statistical indirect association through self-control

Self-control carried a negative statistical indirect association between sense of purpose and IGD symptoms. This pattern is consistent with a statistical link between long-term orientation and everyday behavioral regulation, but the cross-sectional data cannot establish a temporal or causal pathway. Sense of purpose identifies why a goal matters and organizes priorities, whereas self-control concerns whether behavior can be aligned with those priorities when immediate rewards compete with them. This distinction places meaning and behavioral regulation at adjacent but non-equivalent levels of the self-regulatory process (Baumeister, 2018; Converse et al., 2019).

The observed pattern is consistent with evidence from several related literatures. Trait self-control is associated with adaptive behavior across multiple domains (De Ridder et al., 2012), and deficient self-regulation has long been used to explain problematic Internet use (LaRose et al., 2003). More specifically, self-control difficulties have been linked to online gaming addiction among young adults and college students (Safarina and Halimah, 2019; Sumiyana et al., 2022). A recent two-wave study also found that life purpose predicted later self-control among college students, with self-control statistically linking purpose to subsequent academic burnout (Wan et al., 2026). Although academic burnout and IGD symptoms are distinct outcomes, that longitudinal pattern supports the broader proposition that purpose may be expressed through regulatory capacity across college adjustment domains.

The construct checks strengthen, but do not prove, this interpretation. The low HTMT value indicates that the two measures were empirically distinguishable in this sample. Moreover, the indirect association remained evident after excluding the self-control dimensions most similar to goal regulation and when the mediator was restricted to impulse control and healthy habits. Thus, item overlap alone is unlikely to account for the full pattern. Residual conceptual overlap, common-method variance, and omitted third variables nevertheless remain possible. The contribution is therefore not a claim that purpose causes self-control, but evidence that the purpose-IGD association is statistically organized around a distinct self-regulatory correlate that merits longitudinal testing.

### 4.4 Inconsistent mediation and statistical suppression

Our analysis revealed a negative total association and indirect effect alongside a small positive direct coefficient after self-control was included. Opposite signs for the direct and indirect components define an inconsistent mediation or statistical suppression pattern (MacKinnon et al., 2000; Tzelgov and Henik, 1991). Suppressor patterns can clarify how shared variance changes a regression coefficient, but they do not by themselves identify a psychological mechanism (Paulhus et al., 2004). Accordingly, the positive direct coefficient should not be interpreted as evidence that purpose increases IGD symptoms.

The sign reversal instead shows that the bivariate association contains components that operate in different statistical directions once variance shared with self-control is separated. One component is the negative indirect association through self-control. The residual component may reflect measurement characteristics, omitted variables, or aspects of purposeful engagement that are unrelated to behavioral control. For example, gaming research distinguishes achievement, social, and escape motives, which can relate differently to engagement and problematic gaming (Yee, 2006; Király et al., 2015; Laconi et al., 2017). Because motives, gaming frequency, and activity patterns were not assessed, these possibilities cannot be distinguished with the available data.

This suppression pattern refines the interpretation of the overall association. A sense of purpose may coexist with greater engagement in challenging or rewarding activities, but any such tendency cannot be inferred from the direct coefficient alone. What can be concluded is narrower: the negative zero-order association was largely carried by variance in purpose that was shared with self-control. Longitudinal studies should measure gaming motives and behavior at multiple time points to determine whether the residual coefficient is reproducible and whether it predicts adaptive engagement, problematic gaming, or neither.

### 4.5 Moderating role of gender

Gender moderated the association between self-control and IGD symptoms, and the negative slope was stronger among males. This pattern may indicate that variation in regulatory capacity more clearly differentiates IGD symptom levels among male students, a group in which problematic gaming is often more prevalent (Ko et al., 2005; Liu et al., 2022). It is also compatible with dual-process accounts in which addictive behavior reflects an imbalance between reward-driven approach tendencies and inhibitory control (Wiers et al., 2007). The interaction does not show that this imbalance develops differently by gender, but it identifies that possibility as a more precise question for future work.

Several alternatives could produce the same interaction. Male and female students may differ in gaming exposure, symptom variability, preferred genres, gaming motives, or social contexts. Gendered socialization may also shape opportunities to practice or value behavioral restraint (Endendijk et al., 2018). None of these variables was measured, so the stronger male slope cannot be attributed to parenting, peers, motivation, or neurodevelopment. Future studies should test these explanations directly and examine measurement invariance before treating gender as a mechanism rather than a grouping variable.

Adjustment for age left the main coefficients and bootstrap inferences essentially unchanged, and age was not independently associated with self-control or IGD symptoms in the fitted models. Linear age differences therefore did not explain the observed associations within this predominantly young-adult sample. However, the restricted age distribution and the concentration of participants in emerging adulthood limit developmental interpretation (Arnett, 2000). Because the self-control slope remained negative in both gender groups, the findings do not support viewing self-control as relevant only to males. They instead suggest a difference in magnitude that requires replication before gender-tailored implications are considered.

### 4.6 Limitations and future research directions

Several limitations bound our conclusions. First, all focal variables were measured at one time point. The mediation and moderated mediation models therefore partition cross-sectional associations and cannot establish temporal precedence or causal mechanisms (Shrout and Bolger, 2002; Hayes, 2018). Purpose may precede self-control, but stronger self-control could also support progress toward meaningful goals, and IGD-related impairment could affect both constructs. A three-wave design that separates the measurement of purpose, self-control, and later IGD symptoms would provide a stronger test of directionality and reciprocal change.

Second, all variables were self-reported, and Harman’s single-factor result cannot rule out common-method bias. The IGD measure used nine DSM-5 symptom items adapted to a 5-point response format and a 3-month timeframe (Petry et al., 2014). Because all students answered the items without gamer screening, the scores represent symptom severity in the surveyed student population rather than a clinical diagnosis or an estimate among active gamers. Replication should combine a validated Chinese diagnostic or symptom measure with screening for recent gaming and, where possible, behavioral indicators such as play time or platform records.

Third, most participants were recruited from one Beijing university, with only a small number from other institutions. Because the precise number of participating classes and class identifiers were not retained, adjustment for clustering was not possible. Our analyses therefore assume that observations are independent. To the extent that responses within the same class are correlated, this unmodeled clustering may lead to underestimated standard errors and overly narrow confidence intervals, particularly for the smaller second-stage interaction and moderated mediation effects examined here. In addition, multi-site sampling with retained class identifiers and prospectively validated demographic fields is needed to establish whether the pattern generalizes across institutions and regions.

Finally, several measurement-related considerations bound the interpretation of the main findings. First, despite the HTMT and sensitivity results, the very strong bivariate association between self-control and IGD symptoms may partly reflect conceptual or item-content overlap between these constructs; such overlap represents a limitation of the mediation interpretation rather than evidence of a mechanism. Second, because participants’ recent gaming status and gaming frequency were not assessed, the gender interaction may partly reflect differences in gaming exposure between male and female students, a limitation of the moderation interpretation. Future research should compare alternative mediators, including implementation planning, time management, academic self-efficacy, and coping motives, using designs that measure these constructs separately, and should assess gaming status and exposure directly. The practical implications should likewise remain provisional. Evidence on implementation intentions and situational self-control strategies offers testable components for purpose-based or self-regulation interventions (Gollwitzer, 1999; Duckworth et al., 2016), but the current findings do not establish that such programs will reduce IGD symptoms. Randomized or prospective intervention studies should test whether changes in self-control precede changes in gaming-related impairment and whether any benefit differs by gender.

## 5 Conclusion

In this cross-sectional sample of 1,210 Chinese college students, sense of purpose was negatively associated with IGD symptoms at the total-effect level, and self-control carried a significant negative statistical indirect association. The positive direct coefficient after accounting for self-control indicated an inconsistent mediation or suppression pattern. Gender moderated the self-control-IGD symptom association, with a stronger negative slope and conditional indirect effect among males.

These results support further longitudinal investigation of purpose, self-control, and IGD symptoms, but they do not establish that changing purpose or self-control will reduce IGD. Any intervention implications should therefore be treated as hypotheses for prospective evaluation rather than recommendations demonstrated by the present study.

## Data Availability

The original contributions presented in the study are included in the article/supplementary material, further inquiries can be directed to the corresponding author.
